# Evaluation of the Therapeutic Efficacy of the Newly Formulated Drug “Novostron” as an Experimental Basis for Clinical Trials

**DOI:** 10.1021/acsomega.4c11017

**Published:** 2025-08-13

**Authors:** Nailya Ibragimova, Aisulu Kabdraisova, Marina Lyu, Zhanar Iskakbayeva, Arkadiy Krasnoshtanov, Yuliya Tin, Sakhipov Yermek, Seitzhan Turganbay, Roza Karzhaubayeva, Aleksander Ivanovich Ilin, Amirkan Azembayev, Serzhan Mombekov, Saki Raheem

**Affiliations:** † 360736 JSC Scientific Center for Anti-Infectious Drugs, Almaty 050060, Republic of Kazakhstan; ‡ School of Life Sciences, 4921 University of Westminster, 115 New Cavendish Street, London W1W 6UW, U.K.

## Abstract

Chronic wound infections driven by multidrug-resistant (MDR) bacteria continue to challenge modern medicine. This study introduces “Novostron”, an innovative topical iodine-based formulation incorporating dextrin and metal halides designed to overcome the limitations of existing antiseptics, such as volatility and cytotoxicity. The complex’s physicochemical properties were analyzed using infrared (IR) and ultraviolet (UV) spectroscopy, alongside thermogravimetric analysis, confirming its stability and robust iodine retention. Stability assessments revealed minimal iodine loss under optimal refrigerated conditions. In a rat skin wound model, the microbial load was significantly reduced to 1.4 ± 0.5 CFU/cm^2^ within 10 min postapplication, demonstrating rapid antimicrobial action. Wound-healing properties were evaluated on surgically induced wounds in mice, showing accelerated granulation tissue formation and epithelialization within 14 days, supported by histological findings. In infected wound models, combined therapy with “Novostron” and cefazolin enhanced immune responses (IgG and IgM levels), significantly reduced inflammation, and promoted robust tissue regeneration. Additionally, hematological studies revealed decreased leukocytes and thrombocytes, indicating reduced systemic inflammation. These findings suggest the potential of “Novostron” as a therapeutic agent for managing infected and surgical wounds, offering both antimicrobial and wound-healing benefits. However, clinical trials are essential to validate its safety, optimize its application protocol, and establish its efficacy for human use.

## Graphical abstract

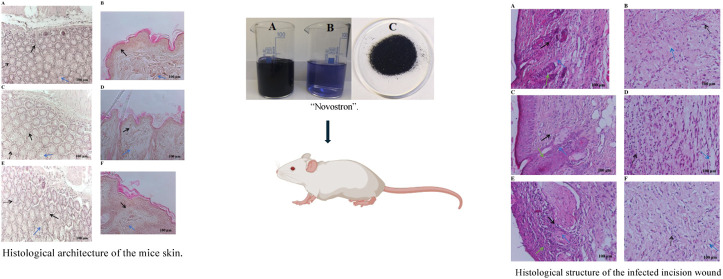

## 1 Introduction

Wound infections are a growing clinical concern, particularly in surgical settings, where hospital-acquired (nosocomial) infections are on the rise. This increase is primarily due to the irrational and unregulated use of antibiotics, often without adequate microbial monitoring, which fosters the development of multidrug-resistant microorganisms.^1^ Chronic wounds and burns are especially vulnerable to infection due to high bioburden and the tendency for biofilm formation, complicating treatment, and healing outcomes.

Among the various antiseptics available for wound management, iodine-based formulations remain highly valued due to their broad-spectrum antimicrobial activity. Iodine has been used for over 150 years and has shown effectiveness against a wide range of microbial strains without contributing to the development of antibiotic resistance.^3,4^ However, the clinical use of iodine is limited by its poor solubility, volatility, and toxicity in its elemental form.^5^

Natural and synthetic polymers have incorporated iodine into complex compounds to address these limitations. Natural carriers such as chitosan, albumin, starch, and glycogen, as well as synthetic polymers like poly­(vinyl alcohol) and polyvinylpyrrolidone, have been used to improve the safety and efficacy of iodine.^2,4–6^ These iodophor formulations reduce the toxic effects of iodine, while enhancing its antimicrobial and antifungal activity. Popular iodophors such as cadexomer iodine (an iodine–dextrin complex) and povidone–iodine (an iodine-polyvinylpyrrolidone complex) are widely used in clinical practice in various topical forms, including ointments, solutions, and dressings.^7^ These agents help reduce bacterial contamination and heal wounds in chronic ulcers, burns, and other injuries.

Povidone–iodine, in particular, is known for its fast and potent antimicrobial effect on planktonic bacteria and biofilms. Despite its effectiveness, there is conflicting evidence regarding its safety in wound healing. Some studies suggest that povidone–iodine may impair healing by exerting cytotoxic effects on human and animal tissue cells, mainly by inhibiting the proliferation of fibroblasts. This has raised concerns about its use in deep or subcutaneous wounds, where tissue regeneration is critical.^8–11^

In contrast, natural polysaccharides like starch and dextrin offer biocompatibility, biodegradability, and nontoxicity, making them promising carriers for iodine.^12^ Polysaccharides play essential roles in biological systems, particularly cellular signaling and recognition. Among polysaccharide-based iodophors, cadexomer iodine stands out for its ability to absorb exudate and release iodine slowly, maintaining prolonged antimicrobial action at infected wound sites.^13^ Studies show that cadexomer iodine enhances epidermal regeneration and reduces ulcer size, outperforming conventional treatments in healing chronic wounds.^14,15^ While cadexomer iodine is particularly adequate in chronic wounds, povidone–iodine remains preferred for treating acute infections.^16^

Further advancements have led to the development of starch cryogels complexed with iodine. These formulations dissolve rapidly and exhibit rapid bactericidal effects, eliminating nearly all bacteria at low iodine concentrations within minutes. Moreover, their superior binding with molecular iodine (I_2_) results in higher iodine retention and stability over time in comparison to other iodine-based solutions. For instance, starch cryogel solutions retain about 73% of iodine content after 1 week at room temperature, significantly more than potassium triiodide (8.5%) and povidone–iodine (2.5%).^17^

Previously, we introduced a novel iodophor formulated as an aqueous solution of a polymer complex incorporating molecular iodine, lithium, potassium, and magnesium halides and α-dextrin. This complex demonstrated exceptional antimicrobial efficacy and significantly enhanced antibiotic susceptibility in various pathogens, including methicillin-resistant *Staphylococcus aureus* (MRSA). Building upon these findings, which highlighted the robust antimicrobial potential of the iodophor complex, the present study seeks to investigate the specific attributes of the formulation “Novostron.” ^18–20^

This study aims to evaluate the antiseptic activity and wound-healing properties of Novostron to determine its suitability for clinical use in treating infected surgical wounds. This investigation seeks to contribute to the development of safer and more effective antiseptic formulations that can improve healing outcomes while minimizing complications associated with traditional treatments.

## 2 Materials and Methods

### 2.1 Chemicals and Reagents

Potassium iodide (KI, 99.8% purity) and iodine (I_2_, 99.8% purity) were purchased from G. Amphray Laboratories (Mumbai, India). Potato starch (analytical grade) was obtained from Birkamidon GmbH (Berlin, Germany). Sodium hydroxide (NaOH, analytical grade) was purchased from AppliChem (Darmstadt, Germany). Poly­(vinyl alcohol) (PVA) with a molecular weight of 31,000 and 99% purity was obtained from Sigma-Aldrich (St. Louis, MO). Pharmaceutical-grade sodium chloride (NaCl, 99.97% purity) was sourced from Salinen Austria AG (Ebensee, Austria). Calcium chloride hexahydrate (CaCl_2_·6H_2_O, 98% purity), magnesium chloride hexahydrate (MgCl_2_·6H_2_O, 98.8% purity), and anhydrous lithium chloride (LiCl, 99.5% purity) were obtained from Penta (Prague, Czech Republic). A 10% (w/v) albumin solution was supplied by Biopharma Plasma (Kyiv, Ukraine).

### 2.2 Preparation of Iodine-Containing Complex for Tropical External Use

Iodophore was synthesized through a simple and controlled reaction process. Hydrolysis of 130 g (802.5 mmol of anhydroglucose units) of starch was carried out in 700 mL of water with the addition of 15 mL of 6N hydrochloric acid, and the mixture was heated at a temperature maintained at or above 88 °C for 30 min. The hydrolysate was then neutralized with 6N sodium hydroxide. The resulting neutralized solution was combined with poly­(vinyl alcohol) (PVA) (3 g, 0.0968 mmol), sodium chloride (4 g, 66.4 mmol), and calcium chloride hexahydrate (2 g, 9.12 mmol). After the mixture was cooled to 43 °C, lithium chloride (4 g, 94.4 mmol) and magnesium chloride hexahydrate (8.2 g, 40.3 mmol) were added, along with 50 mL of a 10% albumin solution. After 15 min, the pH of the mixture was adjusted to 4.5 with sodium hydroxide. After the temperature in the reactor was reduced to 25 °C, the triiodide solution was added. A potassium triiodide solution was prepared by dissolving solid iodine (8.2 g, 32.3 mmol) into a 200 mL aqueous potassium iodide (KI) solution (12.1 g, 72.9 mmol) under continuous stirring.

#### 2.2.1 Characterization and Analysis

UV–Vis absorption spectra were obtained using a UV–Vis Lambda 35 spectrometer (PerkinElmer) equipped with 10 mm quart cuvettes. Measurements were conducted at room temperature across a wavelength range of 190 to 1000 nm to evaluate the optical properties of the iodine-containing complex.

Fourier-transform infrared (FTIR) spectra were recorded using a Nicolet 6700 FTIR spectrometer (Thermo Electron Corporation) in attenuated total reflectance (ATR) mode by employing a horizontal ZnSe accessory. Measurements were performed at room temperature with a spectral resolution of 4 cm^–1^ and an accuracy of ± 0.5 cm^–1^. Each spectrum was acquired from 32 scans ranging from 4000 to 600 cm^–1^. Predried samples were analyzed, and data interpretation was carried out using the OMNIC software suite.

Thermal properties were assessed using an STA 449 F1 Jupiter simultaneous thermal analyzer (Netzsch), which combines thermogravimetry (TG) and differential scanning calorimetry (DSC). Calibration for temperature and sensitivity was performed by using Netzsch reference standards. Samples weighing 3–4 mg were placed in sealed 85 μL alumina crucibles and heated from 28 to 300 °C at a controlled rate of 5 °C/min under a dry nitrogen atmosphere with a 40 cm^3^/min gas flow rate. The analysis provided insights into mass loss and heat flow variations as a function of the temperature.

The iodine content in the complex was determined by using sodium thiosulfate titration and capillary electrophoresis (CE). Iodide ion concentrations were measured by using an Agilent 1600 CE system with a diode-array detector. Samples were prepared in 1.5 mL vials, and a buffer solution at pH 9.3 was used at 25 °C. The iodine–starch complex was treated with an excess of sodium thiosulfate to fully convert iodine into iodide ions. CE was performed using capillaries with a diameter of 50.0 μm and a total length of 56.0 cm while maintaining the cassette temperature at 25 °C. Preconditioning of the capillaries included a 3 min rinse with 0.1N NaOH, followed by a 3 min rinse with buffer solution under a pressure of 900 mbar. The sample was introduced pneumatically at 50 bar for 10 s, with a negative polarity and an applied voltage of −30 kV. Detection was conducted at an absorption wavelength of 226 cm.

#### 2.2.2 Stability Studies

Accelerated Stability Testing: The stability of the iodine-containing complex was evaluated under accelerated conditions. The solutions were dispensed into 50 mL dark glass bottles and stored in a climate chamber maintained at 40 ± 2 °C and 75% ± 5% relative humidity for up to 9 months. At predetermined intervals, samples were analyzed in triplicate to determine the iodine content, potassium iodide levels, and presence of reducing sugars.

Real-Time Stability Testing: The complex solutions were transferred into 50 mL dark glass bottles and stored at ambient room temperature with a relative humidity of 65% ± 5% for up to 9 months. Samples were analyzed in triplicate for iodine content, iodide concentration, and reducing sugar levels at regular intervals.

The stability of the iodine-containing complex under refrigerator conditions was also evaluated. The complex solutions were transferred into 50 mL dark glass bottles and stored at 2 to 8 °C for up to 9 months. Samples were analyzed in triplicate at regular intervals to evaluate the iodine content, potassium iodide concentration, and reducing sugar levels.

### 2.3 *In Vivo* Studies

#### 2.3.1 Animals and Ethics Statement

All experiments were conducted in strict accordance with the guidelines of Directive 2010/63/EU of the European Parliament on the protection of animals used for scientific purposes, as well as the Law of the Republic of Kazakhstan dated March 2, 2022, No. 45-r, “On Responsible Treatment of Animals”. The study protocol was approved by the Ethical Committee for Animal Experimentation of the JSC “Scientific Center for Anti-Infectious Drugs” (Approval No. 12/2023). The animals were housed in five polypropylene cages per cage under standard laboratory conditions, including a temperature of 24 ± 2 °C, a 12 h light/dark cycle, and 35–60% relative humidity. Animals were provided *ad libitum* access to standard rodent diet and water. To ensure acclimatization, all animals were maintained in the laboratory environment for 7 days before the start of the experiments.

#### 2.3.2 Study of the Antiseptic Effect on Intact Skin of Rats

Fifteen 4-week-old Sprague–Dawley rats of either sex, weighing 100 ± 10 g, were randomly divided into three groups: the first group was treated with 0.9% NaCl, the second group with betadine, and the third group with “Novostron.” One day before the experiment, the back hair of each animal was carefully trimmed over an area of approximately 3 cm × 3 cm on both sides to prepare the skin surface for the topical application. Each rat received a single treatment with the corresponding substance. The microbial content of the skin surface was assessed at multiple time points: before treatment and at 10, 30, and 60 min, as well as 3, 6, 9, and 24 h post treatment. Samples from the skin surface were collected and cultured on Tryptic Soy Agar (Sigma-Aldrich) for microbial growth analysis.

#### 2.3.3 Study of the Wound Healing Effect on the Incision Wound Model in Mice

Fifteen 6–8-week-old Swiss albino mice of either sex, weighing 30 ± 5 g, were divided into three groups: the first group was treated with 0.9% NaCl, the second group with betadine, and the third group with “Novostron.” One day before the experiment, the dorsal fur of each animal was shaved and disinfected using a cotton swab soaked in 70% alcohol. An incision wound was created on the back of each anesthetized mouse by making a longitudinal midline incision measuring 2.3 ± 0.2 cm in length. The wounds were sutured on both sides using surgical sutures. The day of wounding was considered day 0. Each mouse then received the corresponding treatment, which was applied externally to the wound area once daily for 7 days.

#### 2.3.4 Study of the Wound Healing Effect on the Infected Incision Wound Model in Mice

Fifteen 6–8-week-old Swiss albino mice of either sex, weighing 30 ± 5 g, were divided into three groups: first group was treated with 0.9% NaCl, second group with cefazolin, and third group with Cefazolin and “Novostron.” One day before the experiment, the animals were shaved using a trimmer, and the skin was depilated with wax strips along the dorsal midline in the cranial direction (level of the interscapular region). After premedication and anesthesia, the surgical field was treated with 70% ethyl alcohol twice, with an interval of 30 s, by wiping the skin with a sterile gauze ball from the center to the periphery. A longitudinal midline surgical incision measuring 2.0 ± 0.2 cm was then made on the prepared surface of the back. Using tweezers, the skin was pulled back to create a subcutaneous sac, where a fragment (∼1.5 cm) of infected suture material was inserted. The suture material had been preincubated with *S. aureus* at an initial concentration of 1 × 10^6^ CFU/mL for 24 h. The skin incision was closed with a continuous suture through both sides. Day 0 was considered to be the day of wounding. The wound surface was treated with “Novostron” once daily for 7 days. Cefazolin was administered intramuscularly once daily for 3 days following the creation of the infected incision wound.

#### 2.3.5 Hematological Studies

At the end of the experiment, the animals were euthanized. Blood was collected via cardiac puncture into EDTA containers^21^ and analyzed using an automatic Z52 VET hematology analyzer (Zytopia Ltd., China) according to the manufacturer’s instructions.

#### 2.3.6 Histological Studies

The tissue surrounding the wound area was excised and fixed in 10% phosphate-buffered formalin for 24 h. The samples were then embedded in paraffin wax and sectioned at 2–3 μm thickness. The histological sections were stained with hematoxylin and eosin (H&E) and examined under a microscope (Carl ZEISS, Germany).

### 2.4 Statistical Analysis

The results are expressed as the mean ± standard deviation (SD). The normality of the data distribution was assessed using the Shapiro–Wilk test. For statistical analysis, a one-way ANOVA followed by a Bonferroni *post hoc* test was performed to compare group means. Statistical significance was set at *p* < 0.05, and all analyses were conducted using GraphPad Prism version 6.0 (GraphPad Software, California).

## 3 Results and Discussion

### 3.1 Preparation of Iodine-Containing Complex for Topical External Use

The preparation of ″Novostron″ was conducted in two stages. The first stage involved adding poly­(vinyl alcohol) (PVA), metal chlorides (Ca^2+^, Na^+^, Li^+^, Mg^2+^), and albumin to a dextrin solution derived from starch hydrolysis. The complex formation was achieved in the second stage by introducing a triiodide solution to the mixture. Upon addition of the triiodide solution, the initially colorless dextrin solution containing metal salts, PVA, and peptide from albumin hydrolysis turned dark blue, a visual indicator of successful complex formation. Figures 1 and 2 show a digital photograph of the iodine-containing complex and a schematic diagram of its preparation.

**Figure 1:**
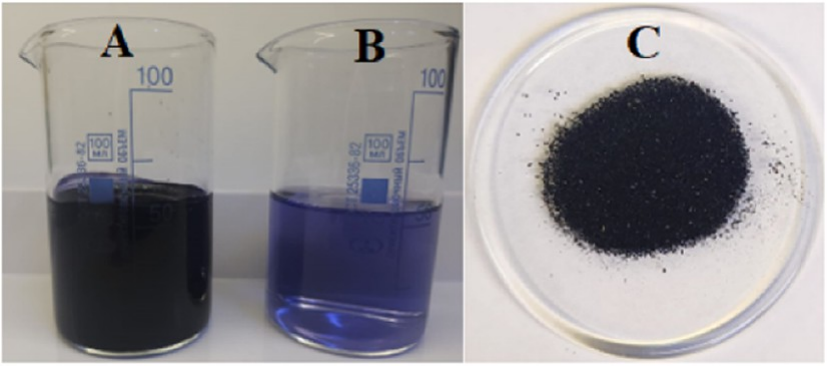
(A) “Novostron”; (B) 0.4% Novostron solution; and (C) dried sample of “Novostron”.

**Figure 2:**
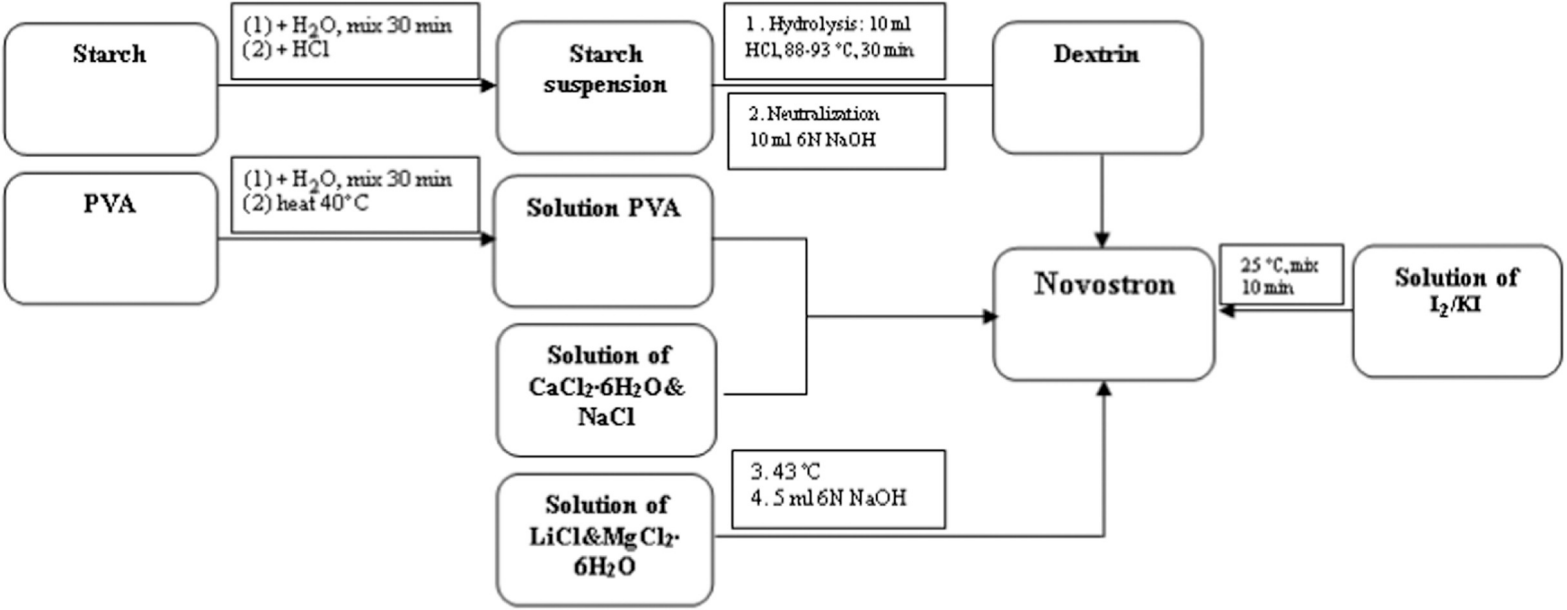
Schematic diagram of the preparation of Novostron.

#### 3.1.1 Characterization and Analysis

The ability of molecular iodine to bind with various natural and synthetic polymers results in the formation of complexes, typically accompanied by a noticeable change in the color intensity or hue. For example, the addition of triiodide to dextrin, derived from starch hydrolysis, changes the reaction medium from colorless to dark violet, signifying the formation of a polymer–iodine complex.

The formation of the iodophor was investigated by using spectrophotometric methods. Potassium triiodide was synthesized by dissolving molecular iodine (I_2_) in a potassium iodide (KI) solution, resulting in the formation of polyiodides, predominantly I_3_^–^. Distinct absorption peaks at 287 and 355 nm confirmed the presence of I_3_^–^. The UV–Vis spectra of the aqueous “Novostron” complex revealed characteristic absorption bands at approximately 290 and 350–360 nm (Figure 3), which are indicative of iodine and iodophor solutions in aqueous environments. It is well established that the absorption maxima at 192–193 and 226 nm correspond to iodide ions (I^–^), whereas the band at 290 nm is attributed to I_3_^–^, and the peak at 350–360 nm is associated with the oxyanion IO^–^. Furthermore, prior studies indicate that solvated molecular iodine (I_2_) exhibits a distinct absorption maximum in the range of 450–460 nm. The synthesized complex exhibits a broad absorption band extending from the ultraviolet to the infrared regions with a central peak observed at 565 nm (Figure 3).

**Figure 3:**
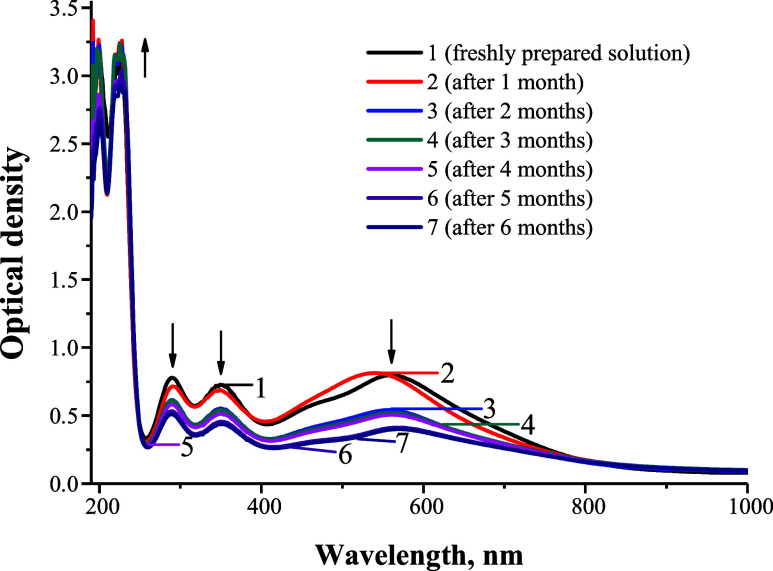
UV–Vis absorption spectra of “Novostron” during 6 months of storage: 1—freshly prepared solution; 2—after 1 month; 3—after 2 months; 4—after 3 months; 5—after 4 months; 6—after 5 months; and 7—after 6 months.

In the visible and UV regions, a gradual decrease in the intensity of absorption bands at 290, 350, and 570 nm was observed, indicating a decrease in the concentrations of I_3_^–^, IO^–^ oxyanions, and the iodide complex, while a simultaneous increase in the optical density at 226 nm, indicates an increase in the content of iodide ions (I^–^). This slow process of iodine reduction lasts for months and is accompanied by a gradual discoloration of the solution.

The FTIR spectrum of “Novostron” is presented in Figure 4. The hydroxyl group absorption band of the complex was detected at 3320 cm^–1^, shifted from the hydroxyl group band of pure dextrin. Peaks in the fingerprint region at 1149, 1074, 1017, and 924 cm^–1^ are associated with C–O bond vibrations characteristic of dextrin, while notable peaks at 1652 and 2931 cm^–1^ were attributed to bound water within the dextrin matrix and stretching vibrations of C–H bonds, respectively. The shift in the hydroxyl group’s absorption band in the complex suggests weak hydrogen bonding interactions between polyiodides and the hydroxyl groups within the dextrin’s spiral structure (Figure 4).^21^ The signal around 1000 cm^–1^ has been recognized as water-sensitive and is associated with intramolecular hydrogen bonding of hydroxyl groups or the plasticizing effect of water.^22,23^ In the spectrum of dextrin, a peak appears at around 997 cm^–1^, and upon the introduction of iodine, the corresponding peak in the complex spectrum shifts to approximately 1017 cm^–1^. This is explained by the formation of a more ordered conformation, i.e., iodine promotes the formation of helical structures.^24^ These spectral features support starch–iodine complexation.

**Figure 4:**
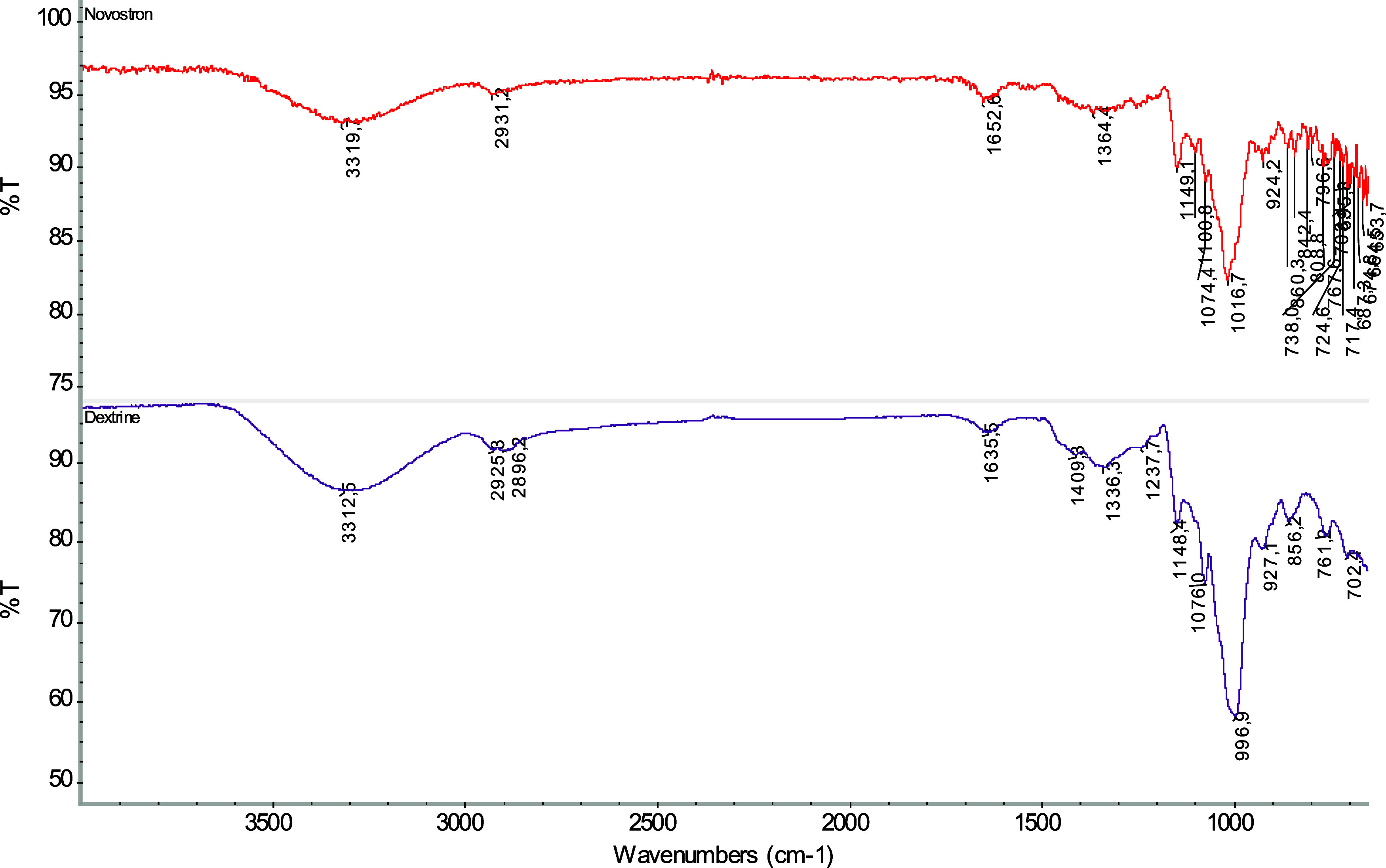
FTIR Spectroscopy of “Novostron.”

The thermogravimetric (TG) curve of the “Novostron” complex (Figure 5) reveals three distinct regions of mass loss. The first region, spanning 28 to 143.8 °C, corresponds to the release of adsorbed iodine and a minor amount of water, with a weight loss of 4.63%, closely matching the iodine content determined by titration. The second stage of mass loss, occurring between 143.8 and 498.6 °C, involves two transitions on the derivative thermogravimetric (DTG) curve. This phase is attributed to the release of iodine triggered by the decomposition of the complex. The final stage corresponds to further degradation of the matrix, demonstrating the stability of the formulation. The iodine content (I_2_) in “Novostron” was quantified using sodium thiosulfate titration and capillary electrophoresis (CE) analysis, both of which corroborated the spectroscopic and thermal findings.

**Figure 5:**
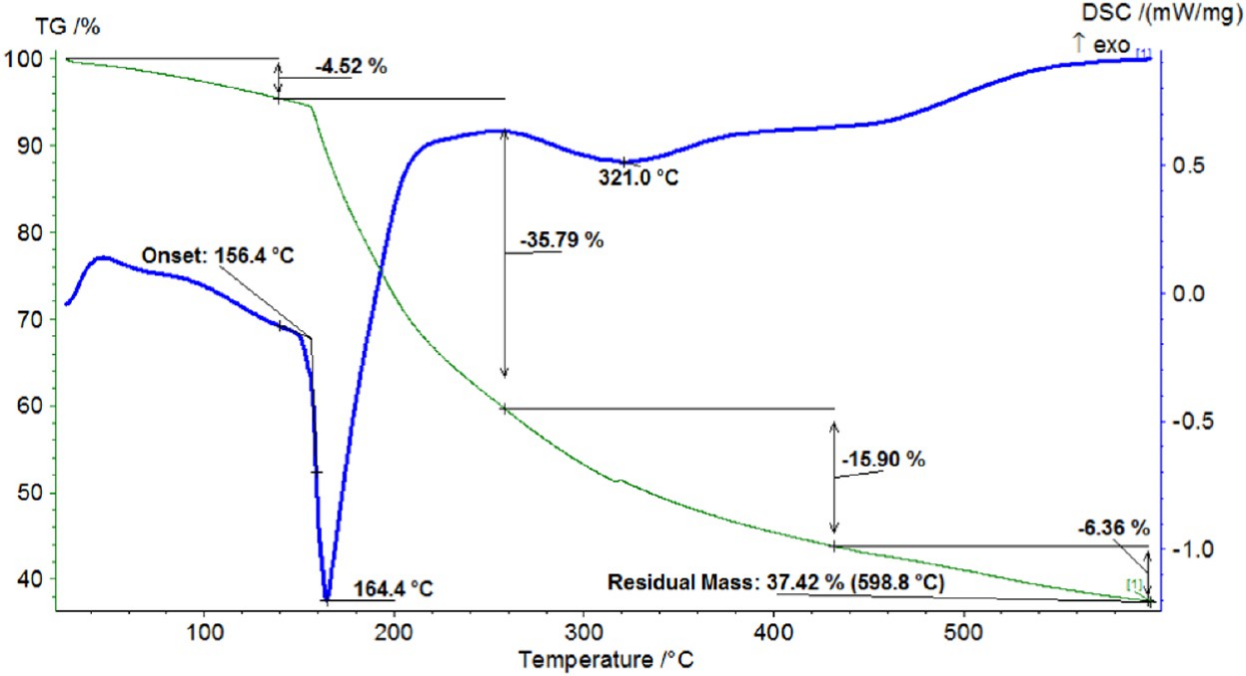
Thermogravimetric analysis (TGA) of “Novostron.”

Therefore, the FTIR data—both the hydroxyl band shift and fingerprint region analysis, collectively provide reliable evidence for the formation and integration of the iodine–dextrin complex. Additionally, DSC analysis revealed a melting temperature of 164.4 °C, corroborating the structural stability of the complex.

#### 3.1.2 Stability Assessment

To facilitate the interpretation of the accelerated stability data, we extrapolated the findings using the ICH Q1A­(R2) guideline, which permits the use of accelerated conditions (40  ±  2 °C, 75  ±  5% RH) to predict long-term stability at ambient temperature (25  ±  2 °C, 60 ±  5% RH). Based on Arrhenius kinetics, which estimates a 2–3-fold increase in the degradation rate for every 10 °C rise in temperature, storage for 3–6 months at 40 °C corresponds to approximately 1–2 years at room temperature.

The obtained accelerated stability data were compared with results from storage at 25 °C and 60% relative humidity (ambient conditions) as well as under refrigerated conditions (5 ± 3 °C). As illustrated in Figure 6, the molecular iodine content in “Novostron” decreased by only 23% over 9 months at 40 °C, while under ambient conditions, it remained highly stable over the same period. Furthermore, refrigerated storage (5 ± 3 °C) showed minimal iodine loss (only 3.8% over 6 months), indicating excellent long-term preservation.

These findings suggest that “Novostron” retains its pharmaceutical efficacy for at least 2–3 years at room temperature and potentially over 5 years under refrigeration, supporting its suitability for both clinical and field applications without immediate cold-chain dependency.

The complex exhibited full water solubility across a typical pharmaceutical concentration range (1–10%). Stability in aqueous solutions was further investigated over 6 months by monitoring spectral characteristics. In the visible and UV regions, a gradual decrease in the intensity of absorption bands at 290, 350, and 570 nm was observed within the first 24 h. This was accompanied by a slight increase in optical density at 226 nm, indicating the gradual transformation of I_3_^–^, IO^–^ oxyanions, and the iodine complex into iodide ions (I^–^). This slow iodine reduction process persisted throughout the 6-month observation period, with discoloration of the solution occurring gradually over time.

These findings underscore the robustness of the complex under varied environmental conditions. Furthermore, the stability of the complex in solution at room temperature was verified by using UV spectroscopy, which confirmed that the key absorption peaks remained stable over time.

**Figure 6:**
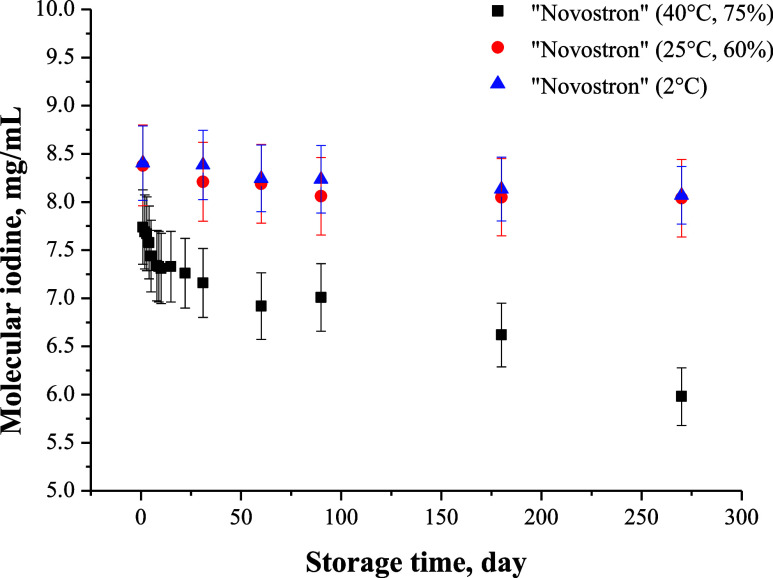
Stability of molecular iodine in the “Novostron” preparation under accelerated and ambient storage conditions over 9 months.

### 3.2 Evaluation of Efficacy

#### 3.2.1 Antiseptic Effect on Intact Skin of Rats

The antiseptic effect of “Novostron” was evaluated through the bacterial count on the skin surface of the rats. The results are presented in Figure 7.

**Figure 7:**
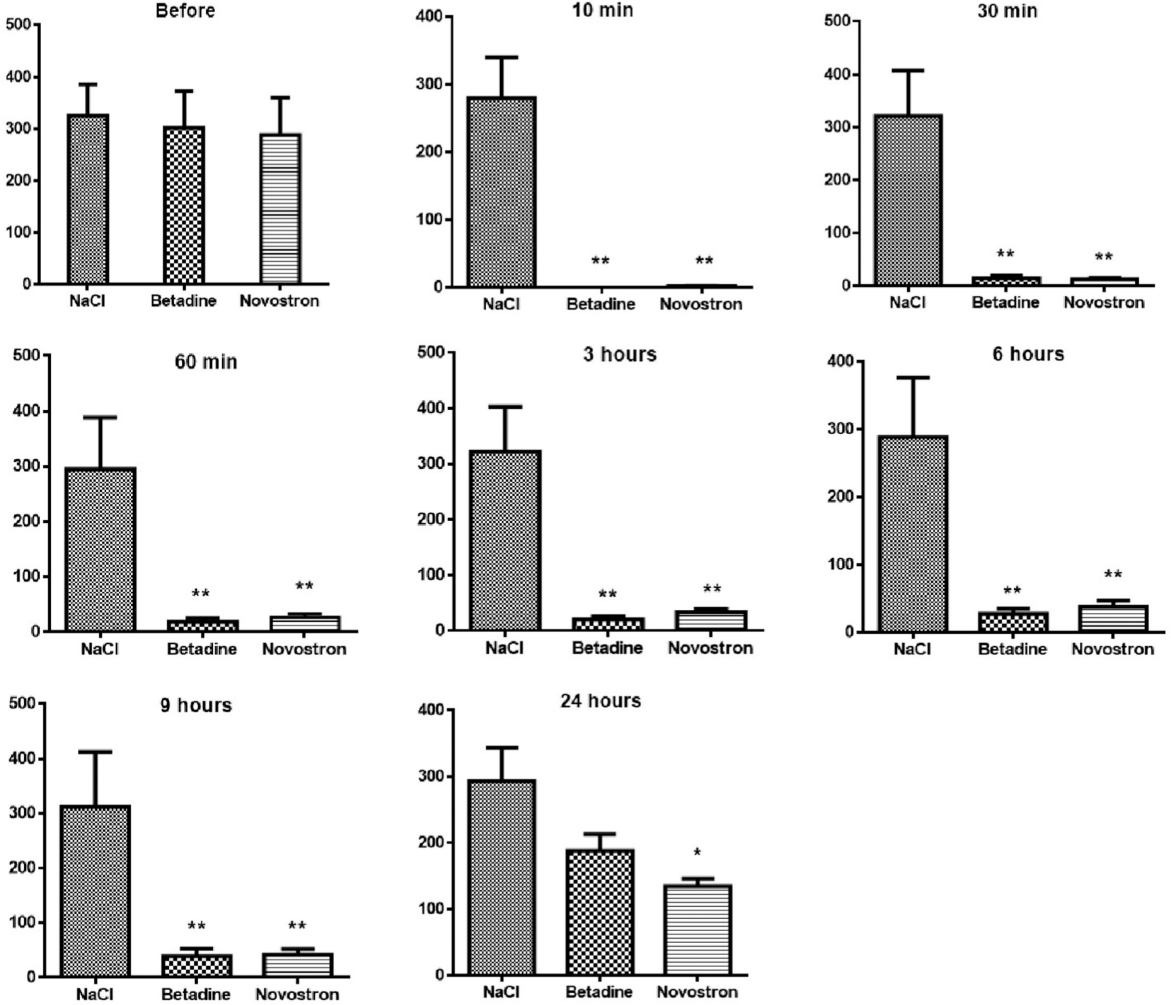
Bacterial count on the skin surface (CFU/cm^2^). Data represent mean ± SD (*n* = 5). *p < 0.05, and **p < 0.01 compared with the NaCl group.

Before treatment, microbial colonization levels were comparable across all groups, with counts of 287.8 ± 72.2 CFU/cm^2^ for “Novostron,” 301.8 ± 70.2 CFU/cm^2^ for Betadine, and 325 ± 60.6 CFU/cm^2^ for the control. After treatment with “Novostron”, a significant reduction in microbial load was observed within 10 min (*p* < 0.001). This effect persisted for 24 h, although the bacterial count gradually increased over time due to the natural restoration of the skin microflora. Betadine exhibited rapid and complete antimicrobial activity, reducing microbial counts to zero within the first 10 min. However, bacterial regrowth was observed over time, with microbial counts reaching 188.0 ± 24.8 CFU/cm^2^ at 24 h. In contrast, the NaCl group maintained a stable microbial count throughout the experiment, reflecting the natural microflora. Both “Novostron” and Betadine significantly reduced bacterial colonization levels compared to the control group. Since betadine exhibits better antimicrobial activity at initial time points, the “Novostron” showed a more substantial antiseptic effect in comparison to betadine (*p* < 0.05) at the later time points, as is seen after 24 h (Figure 7).

#### 3.2.2 Wound Healing Effect of “Novostron” on the Incision Wound Model in Mice

For the study of the wound-healing effect of “Novostron” on a surgical wound model in mice, the hematological analysis was carried out; the results are presented in Figure 8.

**Figure 8:**
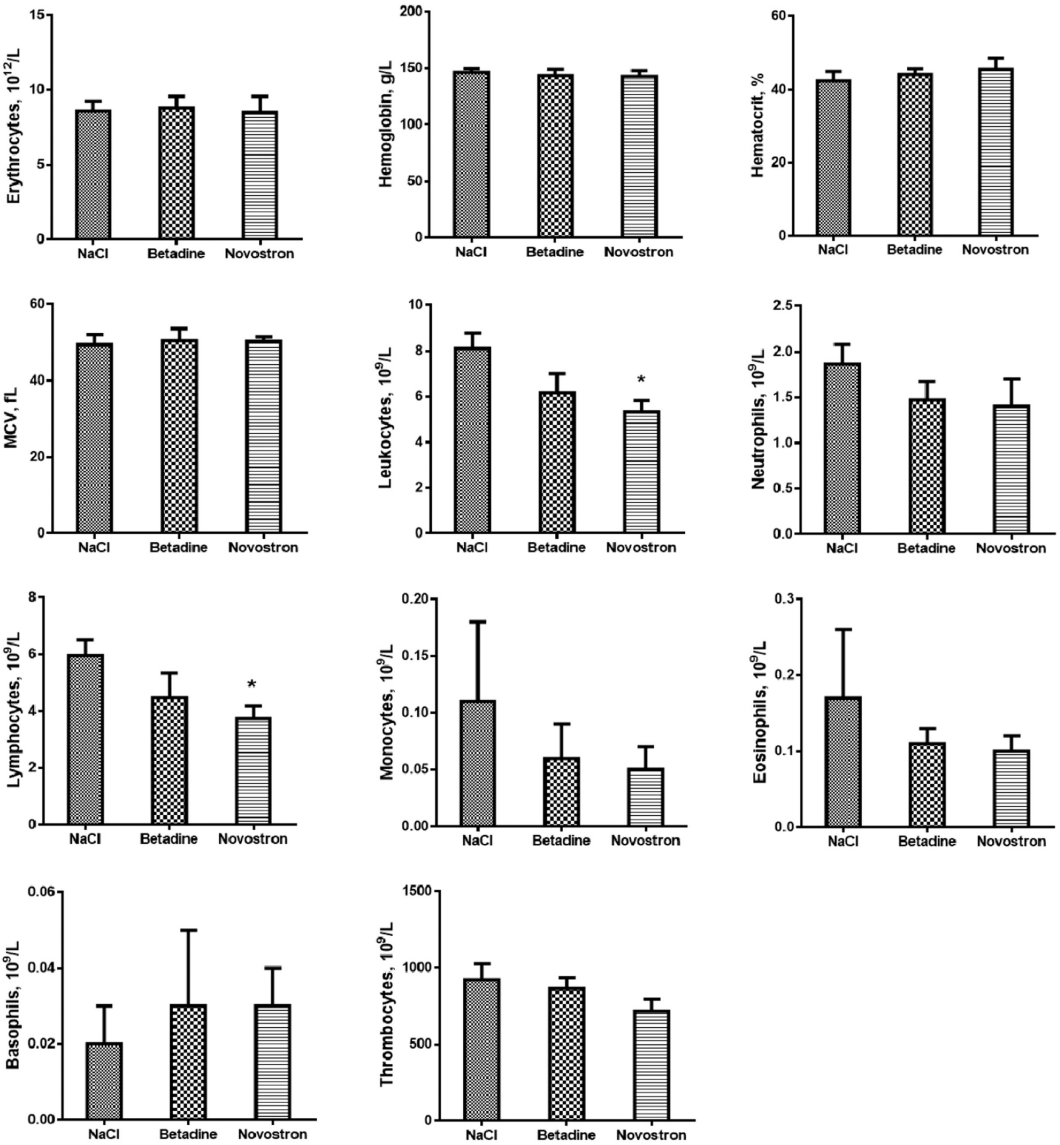
Hematological analysis. Data represent mean ± SD (*n* = 5). *p < 0.05 compared with the NaCl group.

The therapy with “Novostron” showed several changes in hematological parameters, indicating a reduced inflammatory response. A marked decrease in leukocytes and an increase in thrombocytes were observed in the “Novostron” group. These findings suggest diminished system inflammation during the wound-healing process. In the betadine-treated group, lymphocyte reduction was observed, reflecting an immune response characteristic of healing wounds. The decrease of neutrophils in both test groups might be the inhibition of neutrophil migration due to the iodination process, which can interfere with phagocytosis.^25,26^ However, the difference with the NaCl group was minimal and not statistically relevant, which indicates the alleviation of the inflammatory process.

Histological examination of the skin tissue 7 days post treatment revealed distinct differences among the groups, as shown in Figure 9.

**Figure 9:**
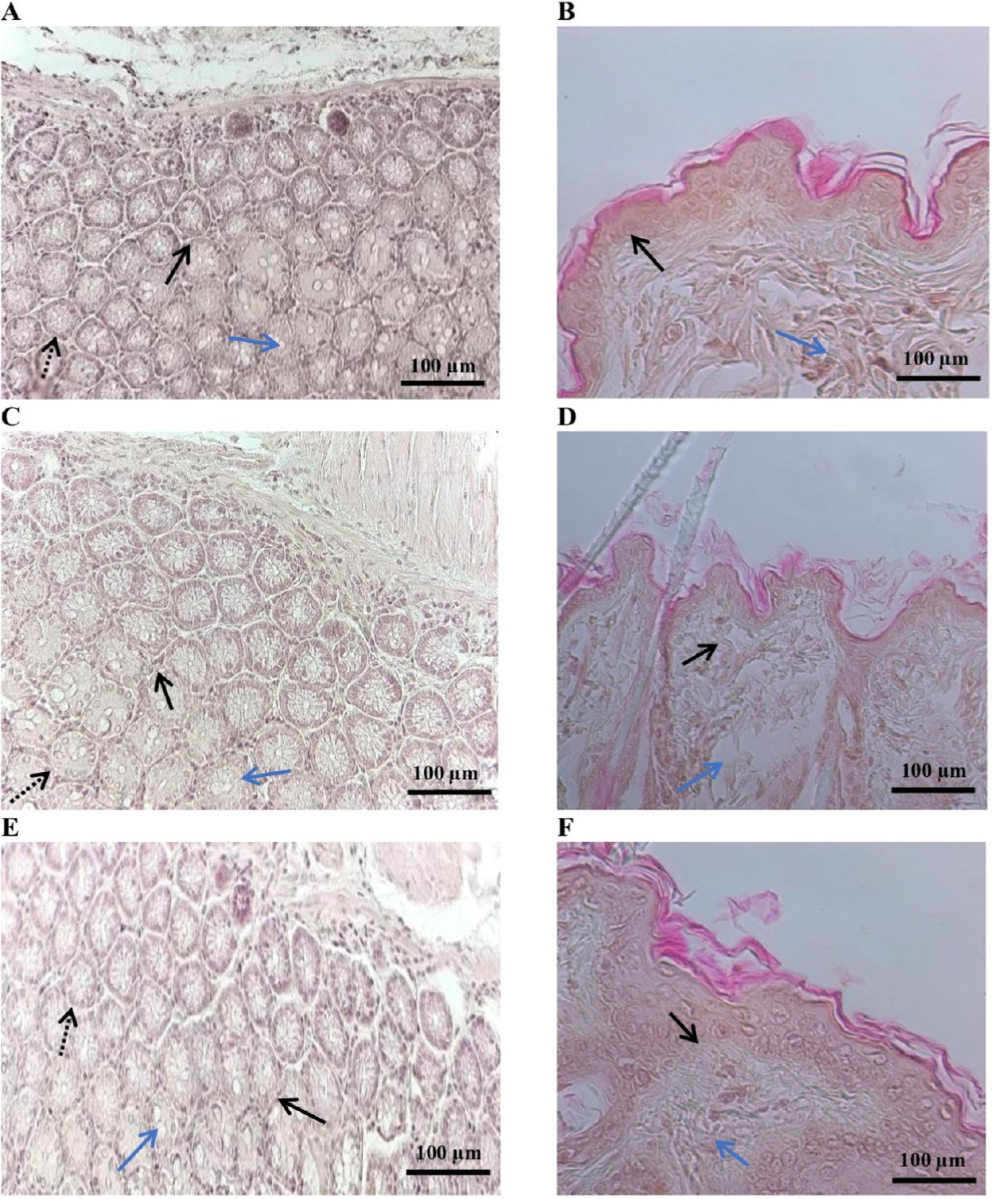
Histological architecture of the mice skin: (A, B) NaCl group; (C, D) Betadine group; and (E, F) “Novostron” group. Black arrow: granulation tissue; black dotted arrow: hair follicles; blue arrow: edema. Scale bar 100 μm (200× magnification).

In the control group, tissue sections exhibited signs of inflammation, including desquamation, epidermal ulceration (Figure 9A), and moderate granulation tissue formation (Figure 9B). These features indicate an ongoing inflammatory response with a limited healing progression. In the betadine-treated group, treated wounds showed desquamation of the epithelial layer (Figure 9C), epidermal hyperplasia, dermal fibroplasia, and moderate subcutaneous edema (Figure 9D). In the “Novostron” group, well-defined circular hair follicles are surrounded by a connective tissue sheath, and some exhibit inflammatory infiltrates (Figure 9E). The epidermis appears hyperplastic with irregular thickening, and the basal layer remains intact (Figure 9F). The “Novostron” therapy also shows the signs of the healing process; however, residual inflammation persisted.

#### 3.2.3 Wound Healing Effect on the Infected Incision Wound Model in Mice

The ″Novostron″ wound-healing effect was evaluated in mice with surgically induced skin wounds aggravated by a *S. aureus* infection. Macroscopic observations of the treated wounds are presented in Figure 10.

**Figure 10:**
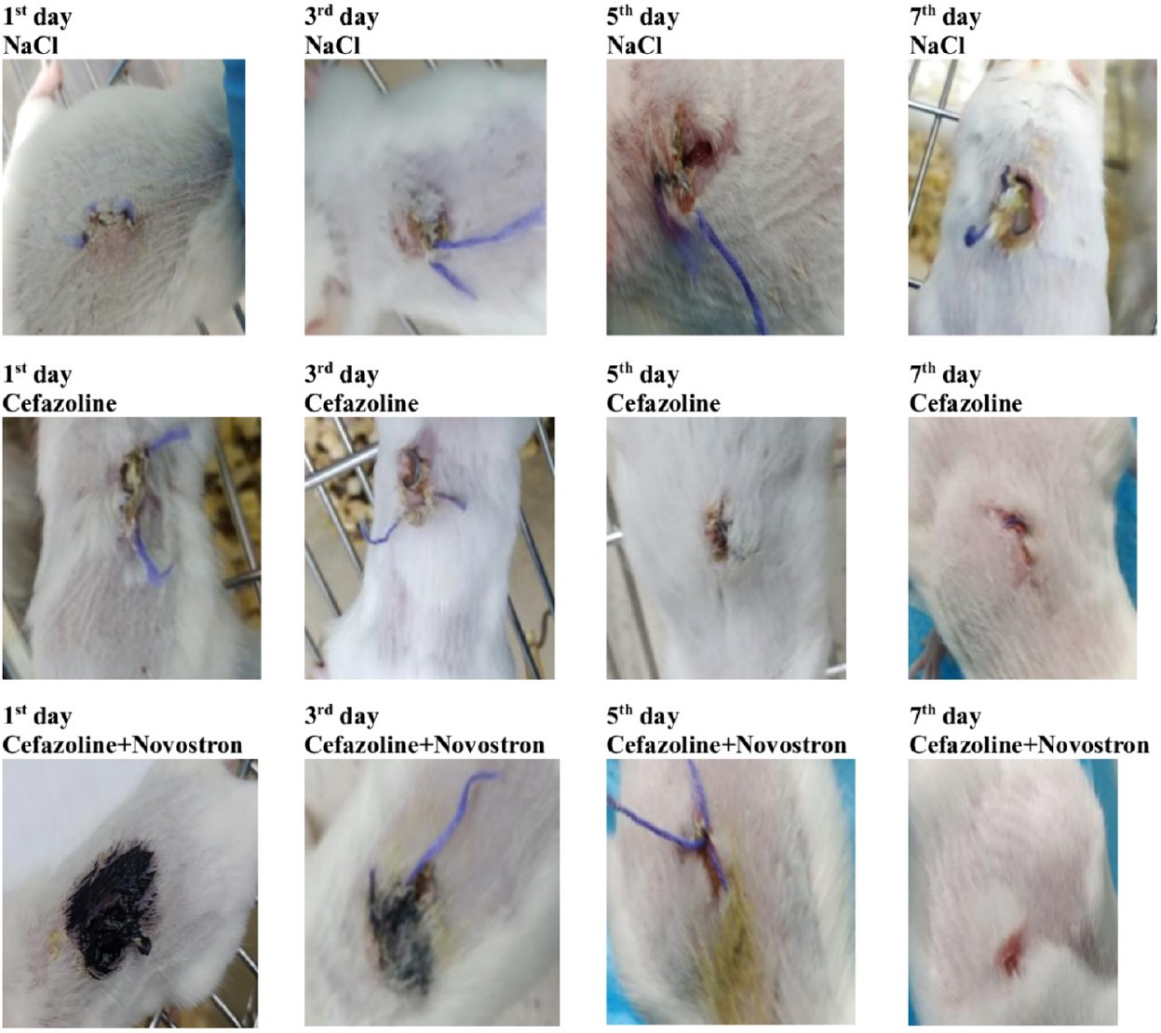
Photographic representation of the infected incision wound on different postincision days. Photograph courtesy of Nailya Ibragimova and Aisulu Kabdraisova. Copyright 2024.

As seen in Figure 10, the health conditions of the animals in all groups remained stable on the first day after the wounding day. From the third day in the NaCl group, the skin condition of the mice worsened, with decreased activity, hunched posture, and clustering behavior being observed. In mice treated with the combined therapy of Cefazolin and “Novostron”, external improvement in their condition was noted by the fourth day, with full recovery of well-being by the seventh day. In contrast, the NaCl animals exhibited a persistent mucous opalescent coating over the infected suture material, which was visible upon extraction due to the absence of the therapy.

Also, the hematological parameters of the animals were studied; the results are presented in Figure 11.

**Figure 11:**
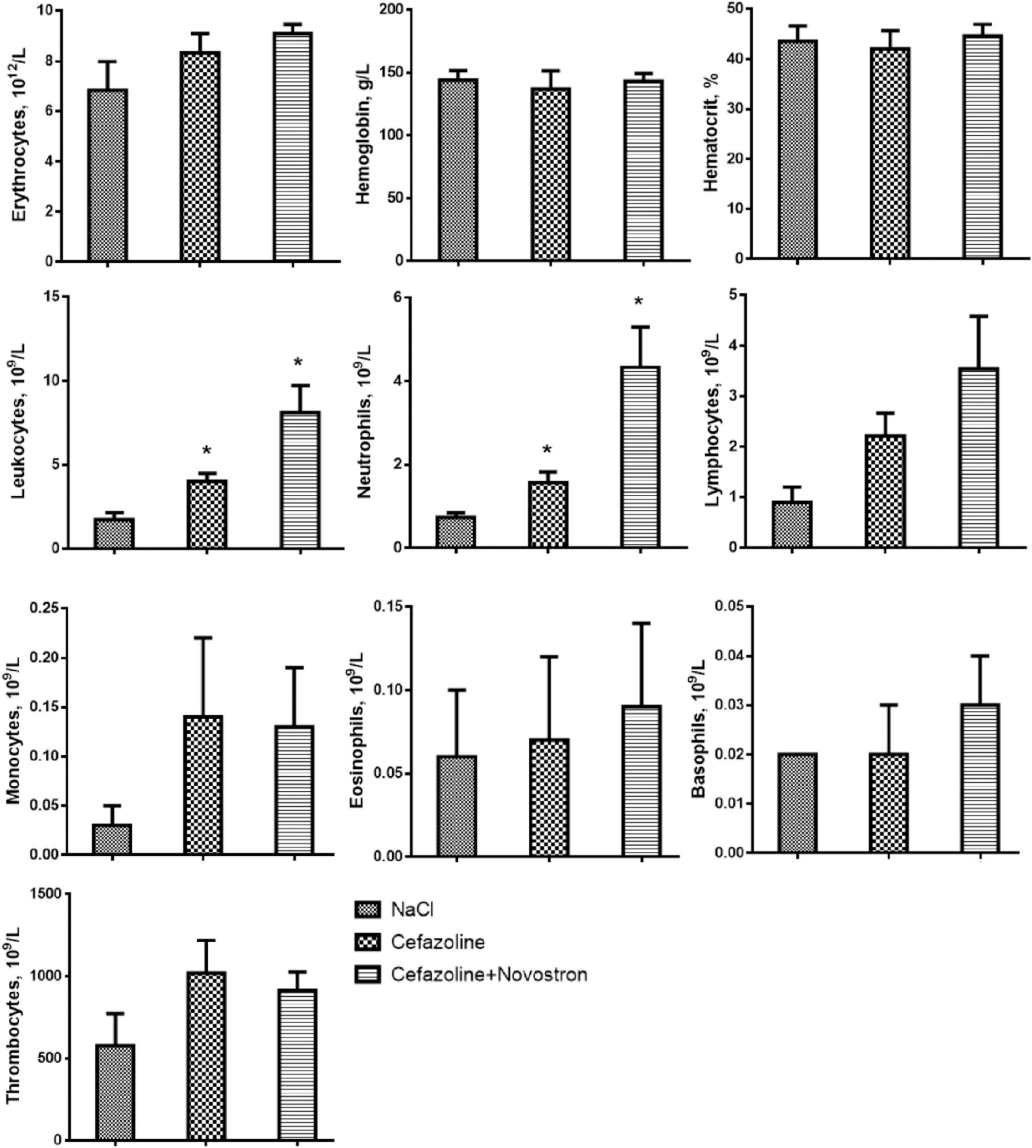
Hematological analysis. Data represent mean ± SD (*n* = 5). *p < 0.05 compared with the NaCl group.

In the NaCl group and the group treated only with Cefazolin, leukopenia was observed. In the group that received combined therapy of Cefazolin and “Novostron,” a significant increase in the leukocytes and neutrophils was observed, indicating positive treatment dynamics. Furthermore, this group also showed increased levels of erythrocytes and monocytes, reflecting enhanced recovery and systemic support during infection resolution (Figure 11).

The degree of the inflammatory reaction was assessed through immunological analysis. The results of measuring the immunoglobulins A, G, and M concentrations in the serum of mice are presented in Figure 12.

**Figure 12:**
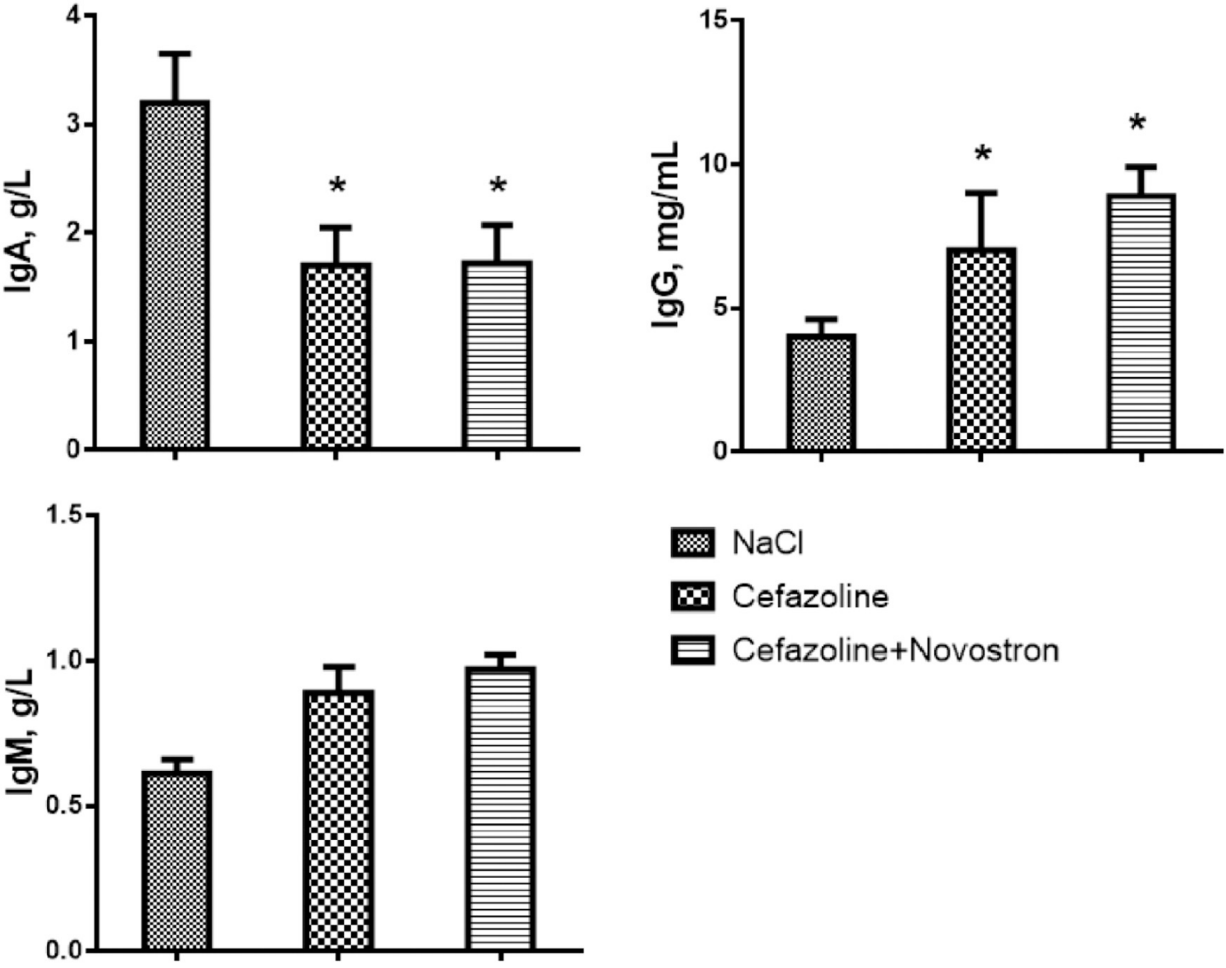
Immunological analysis. Data represent mean ± SD (*n* = 5). *p < 0.05 compared with the NaCl group.

According to Figure 12, a significant increase in the level of IgA was observed in the NaCl group with no therapy, indicating an acute inflammatory process. However, the combined therapy of Cefazolin and “Novostron” showed an increase in the levels of IgG (*p* < 0.05), reflecting a robust activation of humoral immunity and the organism’s enhanced ability to fight infection. A mild increase in IgM levels was observed; however, no statistical relevance of combined therapy and monotherapy was noted compared to the NaCl group.

The findings of the immunological analysis are consistent with those of the histological examination shown in Figure 13.

**Figure 13:**
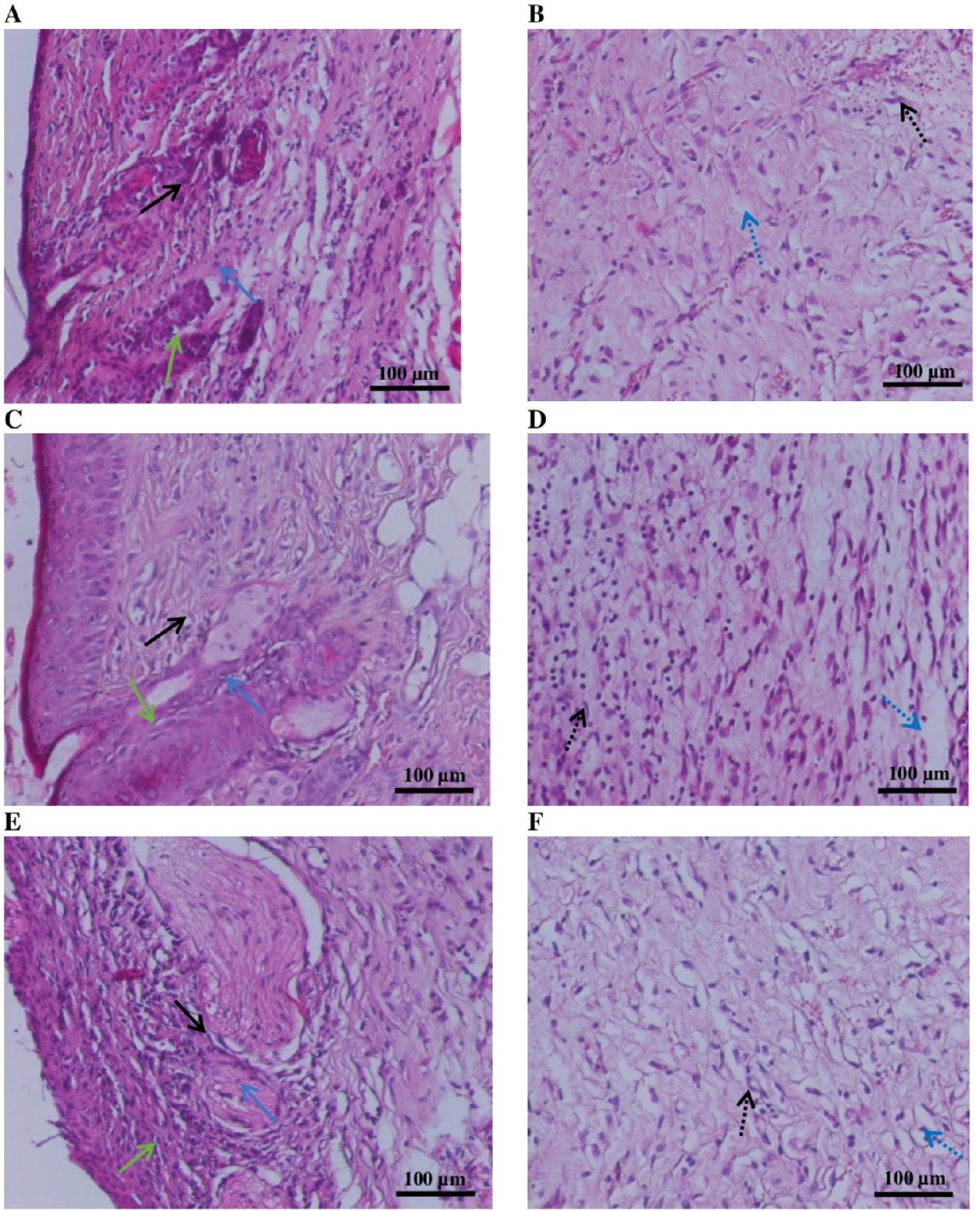
Histological structure of the infected incision wound: (A, B) NaCl group; (C, D) Cefazolin group; (E, F) Cefazolin and “Novostron” betadine. Black arrow: granulation tissue; blue arrow: inflammatory infiltrate; green arrow: interstitial edema; black dotted arrow: fibroblasts; and blue dotted arrow: angiogenesis. Scale bar 100 μm (200× magnification).

In the control group, pronounced signs of inflammation were evident, including interstitial edema, early stage granulation tissue formation, moderate fibroblast proliferation, and angiogenesis (Figure 13A). Coagulative epidermis necrosis extending into the dermis and degeneration of hair follicles were observed (Figure 13B), indicating an unresolved inflammatory process. In the group treated with the combined therapy of “Novostron” and cefazolin, histological sections displayed the formation of mature granulation tissue, active angiogenesis, panniculus adiposus, and intact hair follicles (Figure 13C). The epidermis consisted of 3–4 well-differentiated cell layers, with prominent basophilic nuclei in the basal layer and well-organized collagen fiber bundles in the dermis (Figure 13D). These features suggest accelerated healing and effective tissue remodeling facilitated by the combined treatment. In the cefazolin monotherapy group, differentiation of all epidermal layers, formation of collagen fiber bundles, and individual fibroblasts were noted (Figure 13E,F). However, these changes were less pronounced than in the combined therapy group, indicating a slower and less efficient healing process with monotherapy.

Overall, the results of this work confirmed that “Novostron” positively impacts systemic antibiotic therapy for infected wounds. The formation of a bright pink dermal layer at the suture site during macroscopic and histological examination, as well as activation of blood immune cells and an increase in the levels of IgG, indicate an enhancement of the humoral immunity.^27^ This suggests the promising immunomodulatory effect of the property of the antibiotic potentiator of the new drug “Novostron.”

The anatomy and physiology of mouse skin differ significantly from those of human skin, which poses challenges in translating preclinical findings to clinical practice. Mouse skin is more mobile and contains the panniculus carnosus, a layer of muscle tissue that enables significant contraction of wounds, contributing to a faster reduction in wound size than humans.^28^ Additionally, repair processes in mice are inherently more rapid. Consequently, direct extrapolation of results from rodent models to humans is not feasible without considering these anatomical and physiological differences.

Wound healing proceeds through three overlapping phases: inflammation, regeneration (proliferation), and maturation, during which different areas of the wound may simultaneously exist in various stages. Upon incision, wound healing begins immediately. In surgically sutured wounds, considered standard uninfected wounds in healthy animals, the inflammatory phase is subtle, marked primarily by blood coagulation, with fibrin temporarily binding the wound edges. This is followed by fibroblast migration, ground substance formation, and collagen deposition during the regenerative phase. Granulation tissue, supported by an extensive capillary network, fills the wound, forming a scar formation. By maturation, tissue enzymes degrade excess collagen and temporary matrix, resolving inflammation and forming a stable scar, achieving full tensile strength only after 2 weeks.^29,30^ The resultant scar initially appears red due to abundant vasculature but gradually fades, becoming paler than the surrounding skin as the vascular density decreases.

For an antiseptic to be considered ideal for topical use, it should meet several key criteria: a broad-spectrum antimicrobial activity,^31,32^ high efficacy even in the presence of organic compounds,^33^ promotion of wound healing by curbing inflammation,^34^ good tolerability, affordability, and ease of application.^35,36^

The findings support the idea that “Novostron” possesses properties beneficial for clinical applications, including infection prevention after surgical procedures and accelerated tissue recovery. In addition to its wound-healing and antimicrobial properties, the ability of “Novostron” to stimulate the immune system highlights its potential as an adjunct therapy in cases of compromised immunity, such as diabetic ulcers or chronic wounds. Its broad-spectrum antimicrobial activity, low toxicity, and minimal resistance development position “Novostron” as a promising candidate in modern wound care. Furthermore, the formation of granulation tissue and the absence of prolonged inflammation indicate that “Novostron” prevents infection and actively promotes tissue regeneration. This dual action makes it particularly suitable for managing wounds in high-risk patients, including those with extensive burns or immunosuppression, where infection control and rapid healing are critical.

The stability of “Novostron” under various storage conditions enhances its practicality for widespread use, including in resource-limited settings. The low iodine loss observed at refrigerated and ambient temperatures ensures that the drug maintains its efficacy over time, making it a cost-effective and accessible solution for global health challenges. Moreover, its ease of application and favorable safety profile further strengthen its potential for integration into routine clinical practices.

However, certain limitations of this study must be acknowledged. While the preclinical data on rodents provide valuable insights, the significant anatomical and physiological differences between rodents and human skin must be considered when extrapolating these results. The accelerated healing seen in rodent models may not fully capture the complexity of human wound healing. Additionally, the study did not evaluate the performance of “Novostron” in more complex wound models, such as diabetic or ischemic wounds, which may affect its overall clinical applicability.

Future research should focus on addressing these limitations. Large-scale clinical trials are necessary to evaluate the long-term safety and efficacy of “Novostron” across diverse patient populations. Additionally, studies exploring its mechanism of action, particularly its immunomodulatory effects, would provide deeper insights into its therapeutic potential. Investigating its synergistic effects with antibiotics or other wound-healing agents could also pave the way for combination therapies, enhancing the outcomes for patients with complicated or chronic wounds.

## 4 Conclusions

“Novostron” has shown significant promise as a novel antiseptic and wound-healing agent, effectively enhancing systemic antibiotic therapy, preventing infections, and accelerating tissue regeneration. Its ability to promote granulation tissue formation, activate humoral immunity through elevated IgM and IgG levels, and maintain stability under various storage conditions highlights its practicality and versatility in managing surgical wounds, chronic ulcers, and other high-risk situations. While its broad-spectrum antimicrobial activity, low cytotoxicity, and immunomodulatory properties position it as a strong candidate for clinical applications, further large-scale trials are necessary to confirm its safety and efficacy in humans. With these validations, “Novostron” could be pivotal in advancing modern wound care and improving patient outcomes globally.

## Data Availability

All relevant data are included in the article.
